# Observing the fragmentation of two expanding bullet types and a full metal-jacketed bullet with computed tomography—a forensic ballistics case study

**DOI:** 10.1007/s00414-023-03062-6

**Published:** 2023-07-17

**Authors:** Petteri Oura, Jaakko Niinimäki, Mikael Brix, Eveliina Lammentausta, Timo Liimatainen, Alina Junno, Juho-Antti Junno

**Affiliations:** 1 Department of Forensic Medicine, University of Helsinki, P.O. Box 21, FI-00014 Helsinki, Finland; 2 Forensic Medicine Unit, Finnish Institute for Health and Welfare, P.O. Box 30, FI-00271 Helsinki, Finland; 3 Research Unit of Health Sciences and Technology, Medical Research Center Oulu, Oulu University Hospital and University of Oulu, P.O. Box 8000, FI-90014 Oulu, Finland; 4 Department of Diagnostic Radiology, Oulu University Hospital, Kajaanintie 50, 90220 Oulu, Finland; 5 Department of Archaeology, University of Oulu, P.O. Box 8000, FI-90014 Oulu, Finland; 6 Cancer and Translational Medicine Research Unit, Medical Research Center Oulu, Oulu University Hospital and University of Oulu, P.O. Box 8000, FI-90014 Oulu, Finland; 7 Archaelogy, University of Helsinki, P.O. Box 4, FI-00014 Helsinki, Finland

**Keywords:** Gunshot, Firearm, Expanding bullet, Gelatine, Terminal ballistics, Ballistic imaging, Computed tomography

## Abstract

Computed tomography (CT) may have a crucial role in the forensic documentation and analysis of firearm injuries. The aim of this forensic ballistics case study was to explore whether two types of expanding bullets and a full metal-jacketed bullet could be differentiated by inspecting bullet fragments and fragmentation pattern in CT. Three types of .30 caliber bullets (full metal-jacketed Norma Jaktmatch, expanding full-copper Norma Ecostrike, and expanding soft-point Norma Oryx) were test fired from a distance of 5 m to blocks of 10% ballistic gelatine. CT scans of the blocks were obtained with clinical equipment and metal artifact reduction. Radiopaque fragments were identified and fragmentation parameters were obtained from the scans (total number of fragments, maximum diameter of the largest fragment, distance between entrance and the closest fragment, length of the fragment cloud, and maximum diameters of the fragment cloud). The fragmentation patterns were additionally visualized by means of 3D reconstruction. In CT, the bullet types differed in several fragmentation parameters. While the expanding full-copper bullet Ecostrike left behind only a single fragment near the end of the bullet channel, the soft-point Oryx had hundreds of fragments deposited throughout the channel. For both expanding bullets Ecostrike and Oryx, the fragments were clearly smaller than those left behind by the full metal-jacketed Jaktmatch. This was surprising as the full metal-jacketed bullet was expected to remain intact. The fragment cloud of Jaktmatch had similar mediolateral and superoinferior diameters to that of Oryx; however, fragments were deposited in the second half of the gelatine block, and not throughout the block. This case study provides a basis and potential methodology for further experiments. The findings are expected to benefit forensic practitioners with limited background information on gunshot injury cases, for example, those that involve several potential firearms or atypical gunshot wounds. The findings may prove beneficial for both human and wildlife forensics.

**Supplementary Information:**

The online version contains supplementary material available at 10.1007/s00414-023-03062-6.

## Introduction

Forensic imaging is increasingly utilized in modern forensic investigation [1]. For example, in firearm deaths, three-dimensional (3D) imaging may play a crucial role in the documentation and analysis of gunshot wounds [2, 3]. Computed tomography (CT) in particular has established its role as a routine auxiliary method in many forensic institutes [1]. The main questions in forensic ballistic imaging include gunshot residue, shooting distance, bullet trajectory, and retained bullet fragments [1, 4, 5].

To study terminal ballistics, i.e., the effects of a projectile within a target, a medium simulating human soft tissue is required [3, 6–8]. Conventionally, ballistic soap and ballistic gelatine blocks have served this purpose, and researchers have been required to physically slice open the blocks to visualize bullet tracks and remnant material. However, physical block slicing [9–11] and visualization of remnant material solely by human eye [12] may reduce the accuracy of the findings. In contrast, CT and 3D analysis of intact blocks have proven rapid, accurate, and reliable in creating a permanent record of the bullet track and allowing analysis of various firearms and projectiles [3, 9].

To date, most ballistic imaging studies have centered on bullet trajectory and gunshot cavities [1, 3, 9, 13, 14]. However, retained bullet fragments are commonly encountered in gunshot cases [15]. Fragments may carry pivotal information regarding weapon type and caliber [12]. In this study, we utilized gelatine blocks and CT to study the fragmentation of two expanding bullet types and a full metal-jacketed bullet. Caliber .30 bullets were selected based on their popularity in, e.g., hunting. Comparing the CT findings of a victim’s soft tissues to those obtained in a gelatine test firing may aid forensic practitioners match or exclude a suspected weapon or ammunition type.

Alongside human forensics, ballistic imaging may also be utilized in wildlife forensics [1]. Cases with suspected poaching or other environmental crime may carry sufficient legal interest that forensic imaging of carcasses, for example, will be considered. Ballistic imaging may also have other uses in the field, as there is an ongoing transition towards non-lead bullets in hunting and wildlife management projects [11, 16]. As lead-free ammunition is getting increasingly popular, it would be important to characterize their differences to soft-nose bullets in terminal ballistic behavior.

The aim of this forensic ballistics case study was to explore whether two types of expanding bullets and a full metal-jacketed bullet could be differentiated by inspecting bullet fragments and fragmentation pattern in CT. We hypothesized that bullet fragmentation would follow a distinct pattern depending on bullet type, and these patterns could be detected from CT scans. Identification of these patterns would aid forensic practitioners make inferences about the course of events.

## Materials and methods

### Experimental setting

**Fig. 1:**
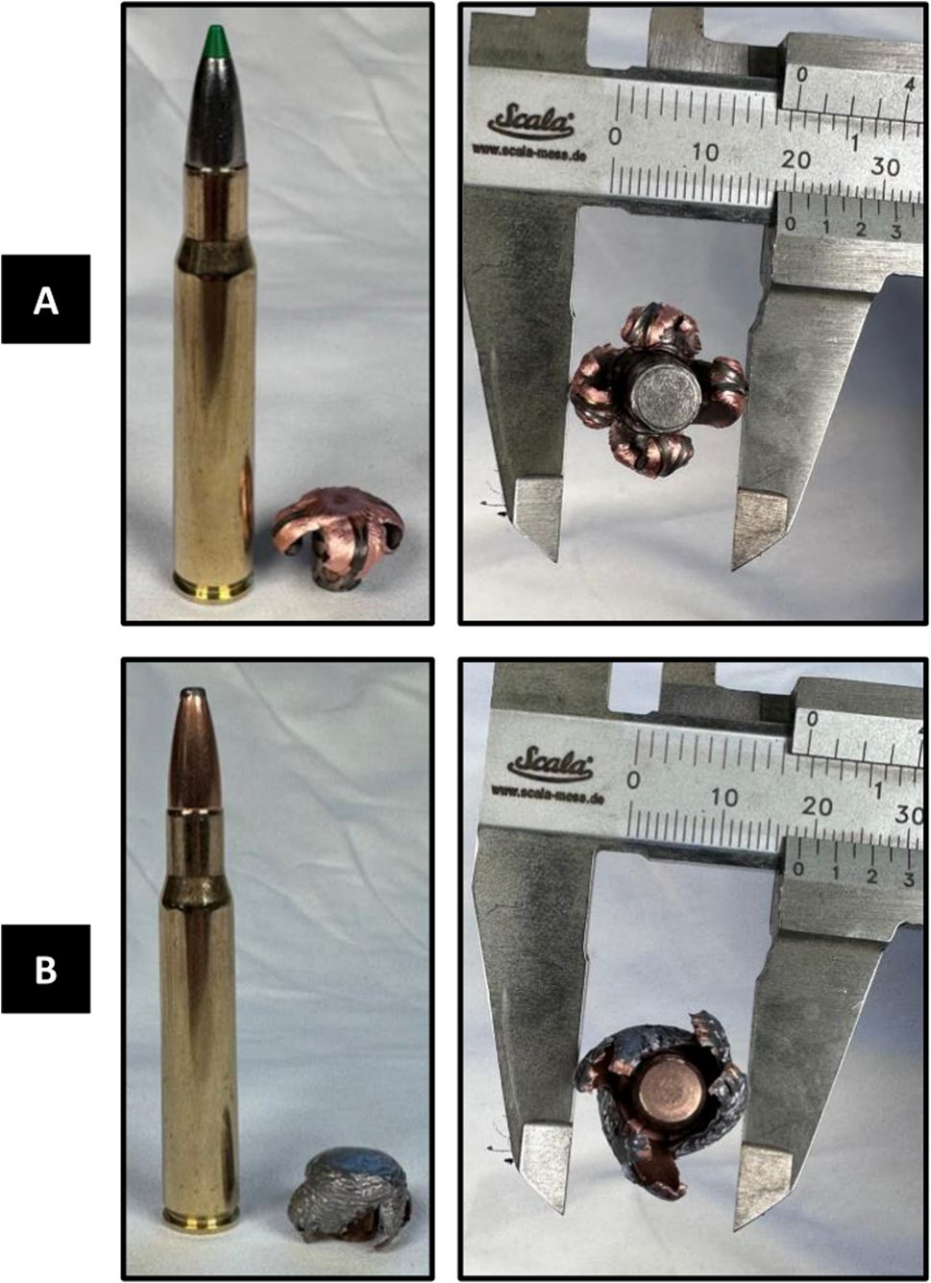
Photographs of the Norma Ecostrike (**A**) and Norma Oryx (**B**) bullets before and after the experiment

Three types of .30 caliber hunting/target bullets were tested in the study: (1) non-expanding full metal-jacketed Norma Jaktmatch (9.7 g); (2) expanding full-copper Norma Ecostrike (9.7 g); and (3) expanding soft-point Norma Oryx (11.7 g) (Norma Precision, Åmotfors, Sweden) (Fig. 1). Caliber .30 was selected based on the popularity of .308 Winchester and .30–06 Springfield; both cartridges are widely available around the world and used for hunting, target shooting, and military purposes. We decided to use Norma ammunition as it is a high-quality manufacturer and has several options for soft-nose and lead-free cartridges in .30 caliber.

Ballistic gelatine (Gelita, Eberbach, Germany) was used to prepare blocks in accordance with the guidance provided by Jussila [17]. Preparation of the gelatine blocks was commenced 48 h prior to the test firing to allow 24-h stabilation at room temperature and 24-h cooling in +4 °C. Blocks with length 500 mm and sides 250 × 250 mm were used in order to ensure that all bullet fragments were retained in the gelatine blocks. Bullet weights were measured before and after the experiment using a calibrated digital scale to an accuracy of 0.01 g.

The test firing was performed using a Tikka M65 Sporter rifle (Tikkakoski, Finland) in the caliber .30–06 Springfield, from a distance of 5 m. An author of the paper (JAJ) with valid firearms license and experience with firearms and ammunition, including the ones used in this study, performed the test firing in a restricted shooting range. A water tank was utilized as a backstop to ensure that bullets could be obtained for further examination after the experiment.

### Computed tomography

The gelatine blocks were scanned in the Department of Radiology, Oulu University Hospital, Oulu, Finland, within 16 h from the experiment. Clinical dual-source CT equipment was used (Somatom Definition Flash, Siemens Healthcare, Forchheim, Germany) with dual-energy CT (DECT) acquisition (rotation time of 0.5 s, pitch factor of 0.3, and collimation of 40 × 0.6 mm). In the DECT acquisition, the tube kilovoltage (kVp) settings were set at 80 kVp and 140 kVp. For the tube operating at the higher kVp setting, an additional 0.4 mm tin filter was used to improve the spectral separation and to mitigate the effects of beam hardening caused by the strongly attenuating projectiles. The reference mAs values were set at 350 mAs and 338 mAs for the 80 kV and 140 kVp acquisitions, resulting in a total volumetric CT dose index (CTDIvol) of 23.4 mGy, and a dose-length product (DLP) of 1295.5 mGy × cm. The data were reconstructed into a voxel size of 0.72 × 0.72 × 0.60 mm using the B30f kernel with the filtered back projection algorithm and a 0.6-mm slice thickness. As the projectiles are strongly attenuating, the extended Hounsfield Unit (HU) scale and metal artifact reduction (MAR) algorithm were used. For the fragment analysis, a mixed DECT image with 0.3/0.7 weighting factors for the low-kVp and high-kVp acquisitions was utilized [18].

### Identification of bullet fragments

**Fig. 2:**
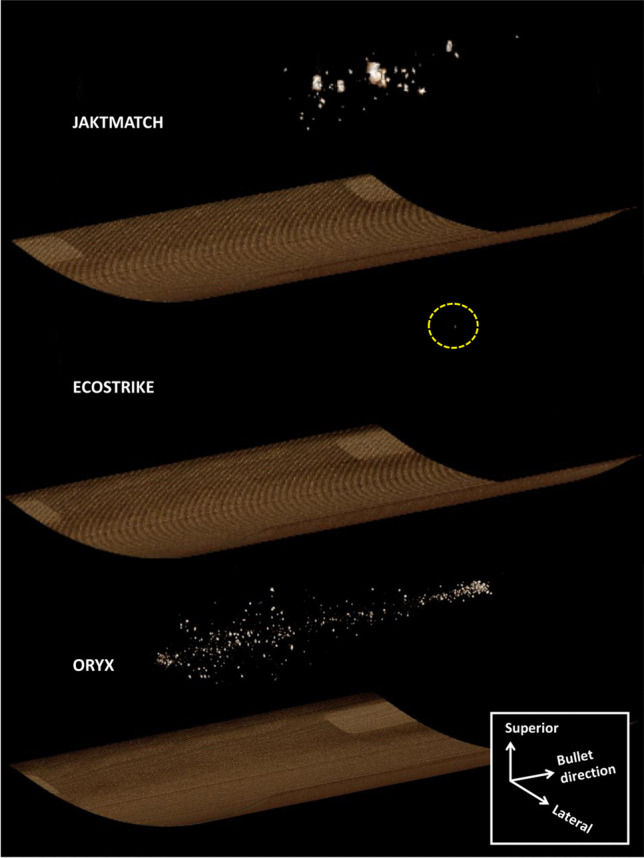
Oblique view of three-dimensional bullet fragment reconstruction based on computed tomography of the gelatine blocks. Dashed circle is used to demarkate the only fragment identified for Ecostrike

**Fig. 3:**
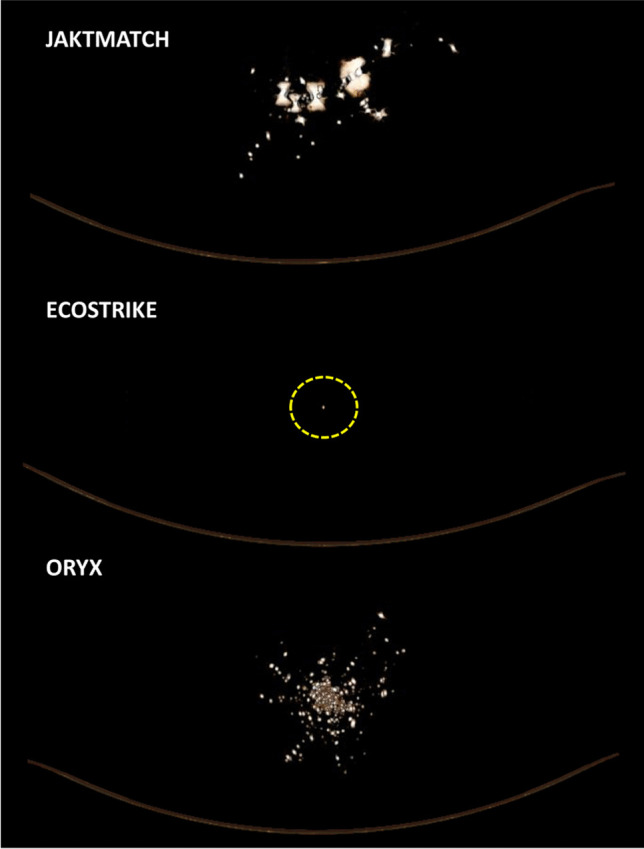
Perpendicular view of three-dimensional bullet fragment reconstruction based on computed tomography of the gelatine blocks. Bullet direction was away from the viewer. Dashed circle is used to demarkate the only fragment identified for Ecostrike

**Fig. 4:**
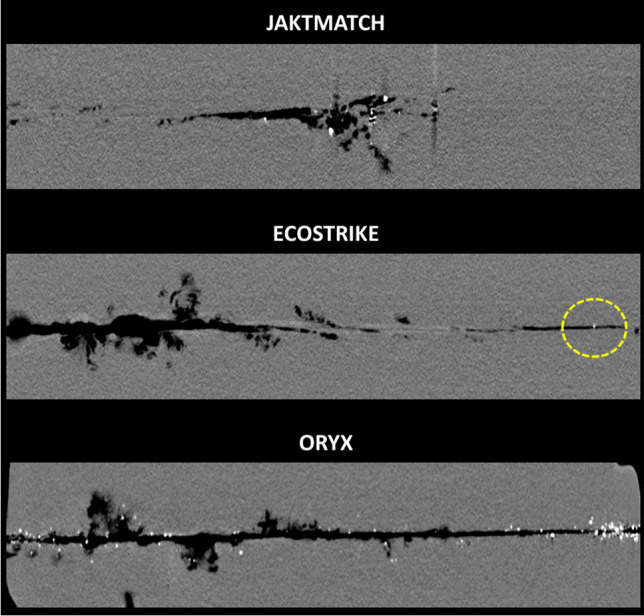
Sagittal computed tomography slice along the bullet channel. Bullet direction was from left to right. Dashed circle is used to demarkate the only fragment identified for Ecostrike

The CT scans were assessed by an author of the paper (PO) in RadiAnt Viewer 2023.1, 64-bit version (Medixant, Poznan, Poland) in DICOM format. The scans were visually evaluated in three perpendicular planes, and a 3D reconstruction of radiopaque fragment material was constructed using the software’s 3D Volume Rendering Tool (Figs. 2, 3, and 4; Supplementary Figures 1—2). In addition, the following numerical parameters were obtained: total number of radiopaque fragments, maximum diameter of the largest fragment in mm, distance between bullet entrance and the closest fragment in cm, length of the fragment cloud along the bullet channel in cm, maximum mediolateral diameter of the fragment cloud perpendicular to the bullet channel in cm, and maximum superoinferior diameter of the fragment cloud perpendicular to the bullet channel in cm (Table 1).

**Table 1:** Bullet weights and fragmentation parameters in computed tomography

	Jaktmatch	Ecostrike	Oryx
Bullet weights			
Original weight (g)	9.69	9.77	11.67
Retained weight (g)	-	9.72	11.46
Weight retention percentage (%)	-	99.5	98.2
Fragments in computed tomography			
Number of fragments (n)	73	1	Hundreds
Maximum fragment diameter (mm)	22.0	4.0	6.5
Distance between the bullet entrance and the closest fragment (cm)	21.3	45.7	0.3
Length of the fragment cloud (cm)	24.7	-	49.5
Maximum mediolateral diameter of the fragment cloud (cm)	13.3	-	8.9
Maximum superoinferior diameter of the fragment cloud (cm)	8.8	-	9.9

## Results

### Test firing

The full metal-jacketed Jaktmatch bullet fragmented at approximately halfway in the gelatine block. Visual observation of the bullet channel indicated that the bullet had rotated around its longitudinal axis in the gelatine before disintegrating. We hypothesized that this was the result of a short shooting distance that did not allow the bullet to stabilize properly. Only fragments of the bullet remained to be examined, as the direction of the bullet shifted in gelatine and it circumnavigated the water tank. It was thus not possible to measure its deformation or weight retention (Table 1).

The full-copper bullet Ecostrike expanded into a 18-mm four-lobed mushroom shape immediately after hitting the gelatine (Fig. 1). Only 0.5% of its weight was lost (Table 1). The bullet channel expanded to a depth of approximately 12 cm, where it reached its maximum diameter of 10 cm. From there, the diameter of the channel decreased and turned into a narrow cavity at a depth of about 30 cm.

The soft-point bullet Oryx left behind a long evenly dilated channel and 1.8% of its weight (Fig. 1, Table 1). After a few centimeters in the gelatine, the bullet expanded into a 15-mm mushroom shape, forming a channel 20 cm long and 10 cm in diameter, which narrowed down into a cavity few centimeters wide approximately in the middle of the block.

### Computed tomography findings

Figures 2 and 3 and Supplementary Figure 1 are 3D reconstructions of the retained bullet fragments based on CT. Figure 4 and Supplementary Figure 2 show sagittal and superoinferior CT slices along the bullet channel. Table 1 presents numerical characteristics of the fragments.

In Jaktmatch, a total of 73 individual fragments of the lead core and the harder jacket alloy were identified. The fragments were largest in size (up to 22.0 mm). The fragment cloud had diameters 13.3 × 8.8 cm perpendicular to the bullet channel. Most fragments existed in the second half of the block.

As for Ecostrike, only one relatively small copper fragment (4.0 mm) was identified; this finding was confirmed by visual inspection of the gelatine block. The single fragment existed towards the end of the bullet channel (45.7 cm from the entrance).

Oryx left behind hundreds of lead and alloy fragments that were identified along the entire bullet channel. The fragments were relatively small in size (up to 6.5 mm). The fragment cloud had diameters 8.9 × 9.9 cm perpendicular to the bullet channel. The dispersion was heaviest in the first half of the block and narrowed down towards the end of the second half.

## Discussion

The aim of this forensic ballistics case study was to explore whether two types of expanding bullets and a full metal-jacketed bullet could be distinguished by inspecting retained bullet fragments and fragmentation pattern in CT scans. As fragmentation appeared to follow a distinct pattern in each case, the findings of this study suggest that CT evaluation may prove useful in forensic cases with limited information on the course of events.

Several of the studied fragmentation parameters were found to differ between the bullet types. While the full-copper Ecostrike left behind only a single copper fragment near the end of the bullet channel, the soft-point Oryx had hundreds of fragments of the core and the jacket deposited throughout the channel. For both expanding bullets Ecostrike and Oryx, the fragments were clearly smaller than those left behind by the full metal-jacketed Jaktmatch. The Jaktmatch fragment cloud had similar mediolateral and superoinferior diameters to that of Oryx; however, Jaktmatch fragments were located in the second half of the gelatine block, and not throughout the block. Although ballistic imaging has been deployed previously in some fragment-related studies [12, 16], comparisons between bullet types have rarely been performed. Moreover, in contrast to 2D imaging modalities, CT may be used to study the 3D distribution of fragments relative to the bullet channel. We were particularly interested in the fragmentation between the two major variants of expanding bullets (Ecostrike & Oryx); the full metal-jacketed bullet was mainly included as a reference.

We believe that our findings are beneficial for future studies combining ballistic gelatine with modern-day forensic radiology. The present methodology is expected to benefit forensic practitioners with limited background information on gunshot injury cases, for example, those that involve several potential firearms or atypical gunshot wounds. Tissue-embedded bullet fragments are commonly encountered in firearm injuries [15], and the fragments may be used to extract valuable information on weapon type and caliber [12]. Comparing the CT findings of a victim’s soft tissues to those obtained in a gelatine test firing may help match a weapon or ammunition type to a particular case. As such, the present findings may prove beneficial for both human and wildlife forensics [1].

The current findings raise the need for several angles of further research. First, other firearms, calibers, and ammunition combinations could be tested in a similar experiment (e.g., hollow-point target, tipped varmint, bonded and non-bonded soft-nose bullets, expanding monobloc bullets). Second, shot distance and/or bullet speed could be varied (e.g., 20, 50, 100, 300 m). Third, bone simulants could be incorporated into the experiment in a way that fragmentation patterns could be investigated in a bone perforation or ricochet scenario.

The main strengths of this study were a standardized experimental setting and 3D imaging modality with clinical equipment and artifact reduction algorithm. The gelatine used here has been concluded to fit the criteria of a valid terminal ballistic simulant [17]. The main limitations of this preliminary study include small sample size (one shot per ammunition), one shooting distance, and potential residual artifacts in CT data. Although 5 m is a short shooting distance, shots from a similar distance with expanding hunting bullets are a reality in big game hunting (e.g., moose hunting in Scandinavian countries) and with full metal-jacketed bullets in urban warfare. Obviously, further studies are needed to confirm the present findings and evaluate the applicability and benefit of fragment identification protocols in routine forensic practice.

In summary, this forensic ballistics case study demonstrated clear differences in bullet fragmentation between a full metal-jacketed, full-copper, and soft-point bullet of .30 caliber. The findings suggest that CT may prove useful in both human and wildlife forensic cases with limited background information on the course of events. This case study provides a basis and potential methodology for further experiments.

### Supplementary information

ESM 1 (DOCX 1155 kb)

## Data Availability

The dataset is available from the corresponding author on reasonable request.
