# Supplementary material for: Observing the fragmentation of two expanding bullet types and a full metal-jacketed bullet with computed tomography—a forensic ballistics case study
Source: Int J Legal Med. 2023 Jul 17;138(2):671–6. doi: 10.1007/s00414-023-03062-6 (PMC10861703; doi:10.1007/s00414-023-03062-6)
Supplement: Supplementary file 1 — (DOCX 1155 kb) [file 414_2023_3062_MOESM1_ESM.docx]

**SUPPLEMENTARY FIGURES**


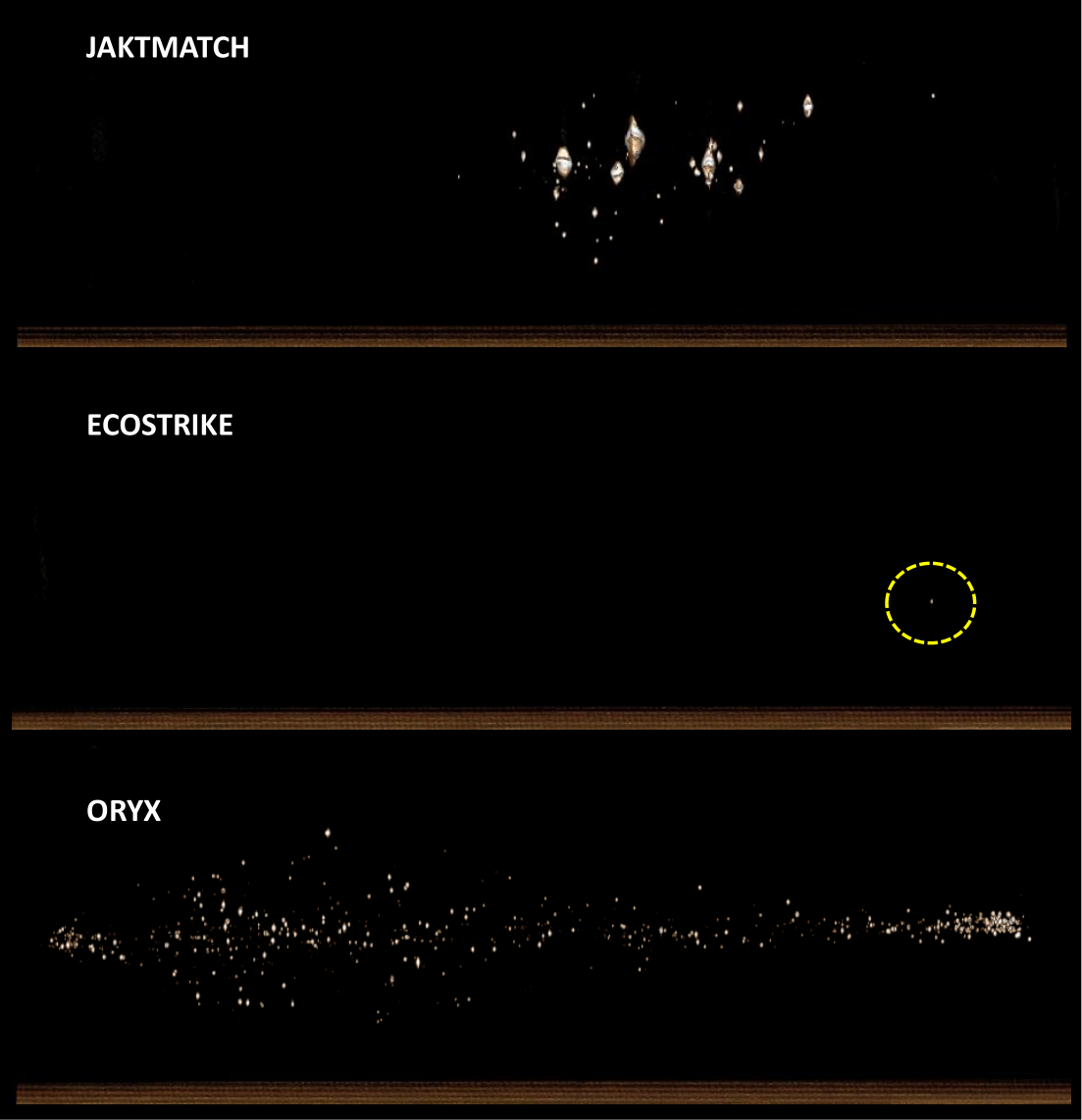


**Supplementary Figure 1**. Lateral view of three-dimensional bullet fragment reconstruction based on computed tomography of the gelatine blocks. Bullet direction was from left to right. Yellow circle is used to demarkate the only fragment identified for Ecostrike.


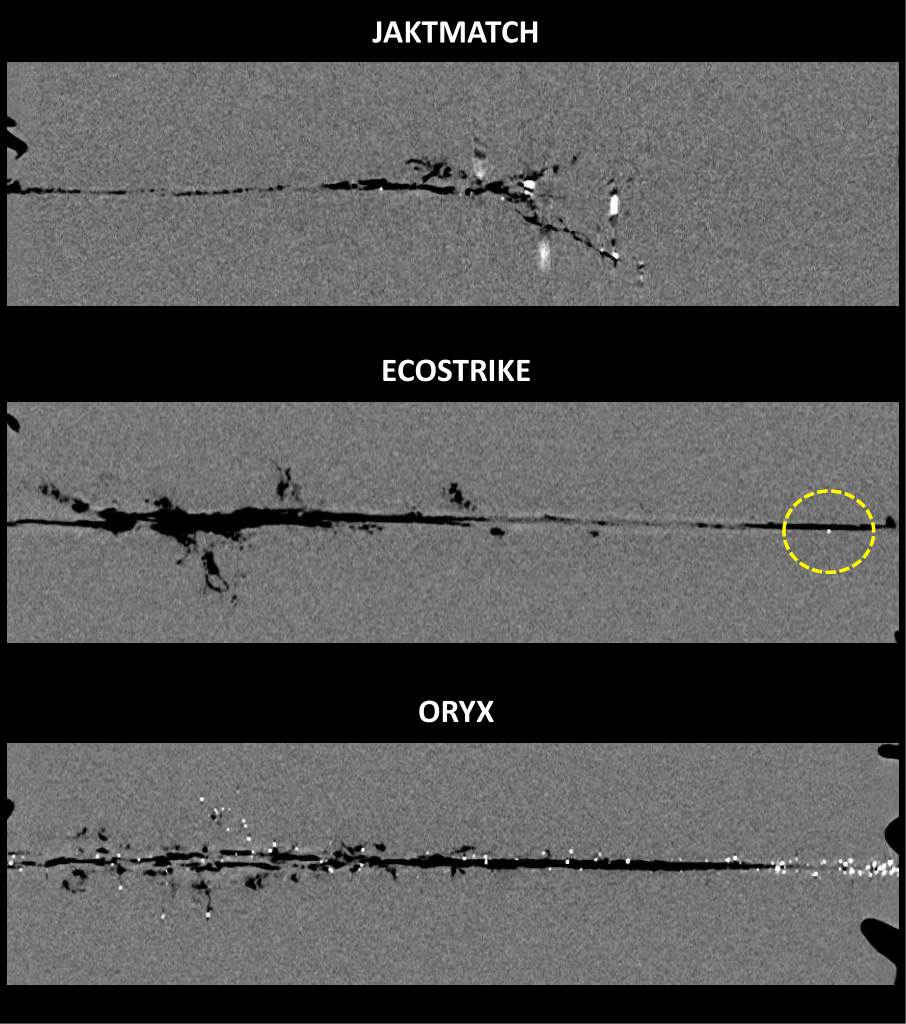


**Supplementary Figure 2**. Superoinferior computed tomography slice along the bullet channel. Bullet direction was from left to right. Yellow circle is used to demarkate the only fragment identified for Ecostrike.
